# A CNN-LSTM-att hybrid model for classification and evaluation of growth status under drought and heat stress in chinese fir (*Cunninghamia lanceolata*)

**DOI:** 10.1186/s13007-023-01044-8

**Published:** 2023-07-03

**Authors:** Dong Xing, Yulin Wang, Penghui Sun, Huahong Huang, Erpei Lin

**Affiliations:** State Key Laboratory of Subtropical Silviculture, Zhejiang A&F University, Hangzhou, 311300 Zhejiang China

**Keywords:** *Cunninghamia lanceolata*, CNN-LSTM, Attention mechanism, Drought stress, Heat stress

## Abstract

**Background:**

*Cunninghamia lanceolata* (Chinese fir), is one of the most important timber trees in China. With the global warming, to develop new resistant varieties to drought or heat stress has become an essential task for breeders of Chinese fir. However, classification and evaluation of growth status of Chinese fir under drought or heat stress are still labor-intensive and time-consuming.

**Results:**

In this study, we proposed a CNN-LSTM-att hybrid model for classification of growth status of Chinese fir seedlings under drought and heat stress, respectively. Two RGB image datasets of Chinese fir seedling under drought and heat stress were generated for the first time, and utilized in this study. By comparing four base CNN models with LSTM, the Resnet50-LSTM was identified as the best model in classification of growth status, and LSTM would dramatically improve the classification performance. Moreover, attention mechanism further enhanced performance of Resnet50-LSTM, which was verified by Grad-CAM. By applying the established Resnet50-LSTM-att model, the accuracy rate and recall rate of classification was up to 96.91% and 96.79% for dataset of heat stress, and 96.05% and 95.88% for dataset of drought, respectively. Accordingly, the R^2^ value and RMSE value for evaluation on growth status under heat stress were 0.957 and 0.067, respectively. And, the R^2^ value and RMSE value for evaluation on growth status under drought were 0.944 and 0.076, respectively.

**Conclusion:**

In summary, our proposed model provides an important tool for stress phenotyping in Chinese fir, which will be a great help for selection and breeding new resistant varieties in future.

## Introduction

### Background

 With global warming, drought and extremely high temperature events have become more and more frequent in southern China. Higher temperatures and less rainfall caused by global warming will lead to extreme weather events (e.g., droughts and high temperatures) in the future [1]. Many studies have indicated that drought and high temperature were important environmental stresses affecting tree growth, development and distribution, and even forest ecosystems and biogeographic processes [2–4]. *Cunninghamia lanceolata* (Chinese fir), an evergreen coniferous tree mainly distributed in southern China [5], is one of the most important timber trees with great commercial value due to its fast growth rate, high yield, high quality and pest resistance [6, 7]. However, frequent occurrences of extreme drought and high temperature events are becoming great risks for growth and biomass production of Chinese fir [8–10]. Therefore, it is an important subject to select or breed drought and high temperature resistant varieties for breeders of Chinese fir. Although there was a long breeding history, artificial selection on stress-resistant varieties of Chinese fir still relies on expert visual observation and physiological measurements [8, 11], which are time-consuming, labor-intensive, costly and prone to human error. To develop an efficient, automated and accurate method for evaluation and classification on growth status is of great significance to stress-resistant varieties selection and breeding in Chinese fir.

Computer vision-based phenotyping provides a simple, rapid, and highly automated method for evaluation and classification on plant physiological and growth status [12–14]. Especially the emergence of convolutional neural networks (CNN) makes plant phenotyping under different stresses more and more efficient and automated. CNN and CNN-based methods have been widely applied in related works. For instance, Lin et al. [15] proposed a semantic segmentation model based on CNN to detect the powdery mildew on cucumber leaf images at pixel level, achieving an average pixel accuracy of 96.08%. Selvam and Kavitha classified leaf image into three categories namely healthy, disease and leaf burn in lady finger (*Abelmoschus esculentus*) with a custom CNN architecture, which achieved 96% classification accuracy [16]. And, deep neural network was applied to detect wheat head in real time with average precision of 94.5% [17].

Although single CNNs have great performance in classification and segmentation of images, they are not appropriate for images from dynamic systems, such as time-series image datasets acquired from the whole growth period. For plant growth, temporal information, such as growth patterns, is one of the key factors in understanding plant resistant capacity to stress and should be taken into account. This problem can be solved by using recurrent neural networks (RNN). In particular, long short-term memory (LSTM) has a very good performance in analyzing dynamic information [18–20]. Conjunction of CNN and LSTM could integrate spatial and temporal information from processing signals to help predict plant growth status more precise. CNN-LSTM predictive methods have been widely applied in the field of botany research and agriculture. Namin et al. [21] combined CNN and LSTM for the classification of various *Arabidopsis* genotype, Abdalla et al. [22] applied Inceptionv3-LSTM framework to diagnose the nutritional status of oilseeds in the field. Turkoglu et al. [23] proposed Multi-model LSTM-based Pre-trained Convolutional Neural Networks (MLP-CNNs) as an ensemble majority voting classifier for the detection of plant diseases and pests. Chang et al. [24] successfully constructed and trained deep-learning models based on the deep convolution neural network (DCNN) and LSTM for the nitrogen nutrition diagnosis of muskmelon.

On the other hand, traditional deep neural networks often failed to accurately locate and extract the discriminative regions of interest when processing images, especially for the plant images, which greatly affects the classification and detection accuracy of images [25] [26]. The attention mechanism (AM) in deep learning, which is similar to visual attention of human, could selectively focus on the target area of interest and ignoring the irrelevant regions of the image [26–28], and then invests more attention resources in the target area to improve the accuracy image processing. Thus, the attention mechanism has been used to improve and optimize the deep neural network architecture. Zhang et al. [29] used the attention mechanism in natural language processing, which greatly improved the translation accuracy. Zhang et al. [26] successfully classified flower images by embedding spatial attention module and channel attention model in Xception structure. Zeng et al. [30] proposed a Self-Attention Convolutional Neural Network (SACNN), which effectively extracts features of disease spots to recognize crop diseases.

Many deep neural models have been proposed to classify and evaluate the growth status of diverse broad-leaved plants [31–33]. As a conifer tree, Chinese fir has thin, needle-like and waxy leaves, which are completely different from broad leaves. The phenotypic changes of Chinese fir plants under stresses, such as changes in needle color and degrees of leaf wilting, are distinctly different to those broad-leaved plants. Those deep neural models fitting for broad-leaved plants are not suitable for needle-leaved tree, such as Chinese fir. It is still a big challenge to classify and evaluate the growth status of Chinese fir under different stresses through deep neural networks. In addition, the model should overcome the interference caused by the appearance similarity of different status. Considering the great importance of Chinese fir in timber industry of China, and the potential negative impact of climate change and global warming on production of Chinese fir, it is urgent and meaningful to develop image-based methods for classification of growth status under drought and heat stress to facilitate breeding programs. To address the above issues, a hybrid deep learning network CNN-LSTM-att was designed to classify and evaluate the growth status of Chinese fir seedlings under drought and heat stress. The detailed contributions are stated as follows:

A) Since there was no publicly available image dataset of Chinese fir seedlings under drought stress or heat stress, we created two image datasets based on drought and heat treatment of Chinese fir seedlings, respectively. And, the growth status of Chinese fir seedling in each image was also manual scored with a value between 0 and 1.0.
B) We combined CNN and LSTM to learn and classify the temporal and spatial information of growth and damage degree of Chinese fir seedlings under drought and heat stress, respectively. Compared with base CNN network, the classification accuracy has been greatly improved.
C) We embedded the attention mechanism into the backbone network of the CNN-LSTM to enhance the feature extraction ability of the network.
D) We proposed a CNN-LSTM-att model to classify and evaluate the growth status of seedlings under drought and heat stress, which provides a useful tool for stress phenotyping on a large number of germplasms in Chinese fir.

## Materials and methods

### Plant materials and stress treatment

To create the image datasets of Chinese fir under drought and heat stress, the seedlings were treated by artificial drought and heat stress, and images were then taken at different time point. The seeds of Chinese fir, obtained from an orchard in Kaihua forest farm of Zhejiang Province, China, were used to cultivate the seedlings in a green house. The seedlings with about 20 cm in plant height were subjected to heat and drought stress, respectively. For the heat stress, 55 seedlings were selected and placed in a growth chamber, and the environment was set as follows: temperature 43 ℃, relative humidity 80% and Photosynthetic Photon Flux Density (PPFD) 200 µmol. m^− 2^. s^− 1^. And, the treatment was performed in the growth chamber for 30 h. For the drought treatment, 45 seedlings were used, and the drought condition was simulated by irrigation with 20% PEG6000. 30 ml of 20% PEG6000 solution was irrigated to each seedling every 7 days, and the treatment was performed in a greenhouse for 35 days.

## Image acquisition and annotation

**Fig. 1:**
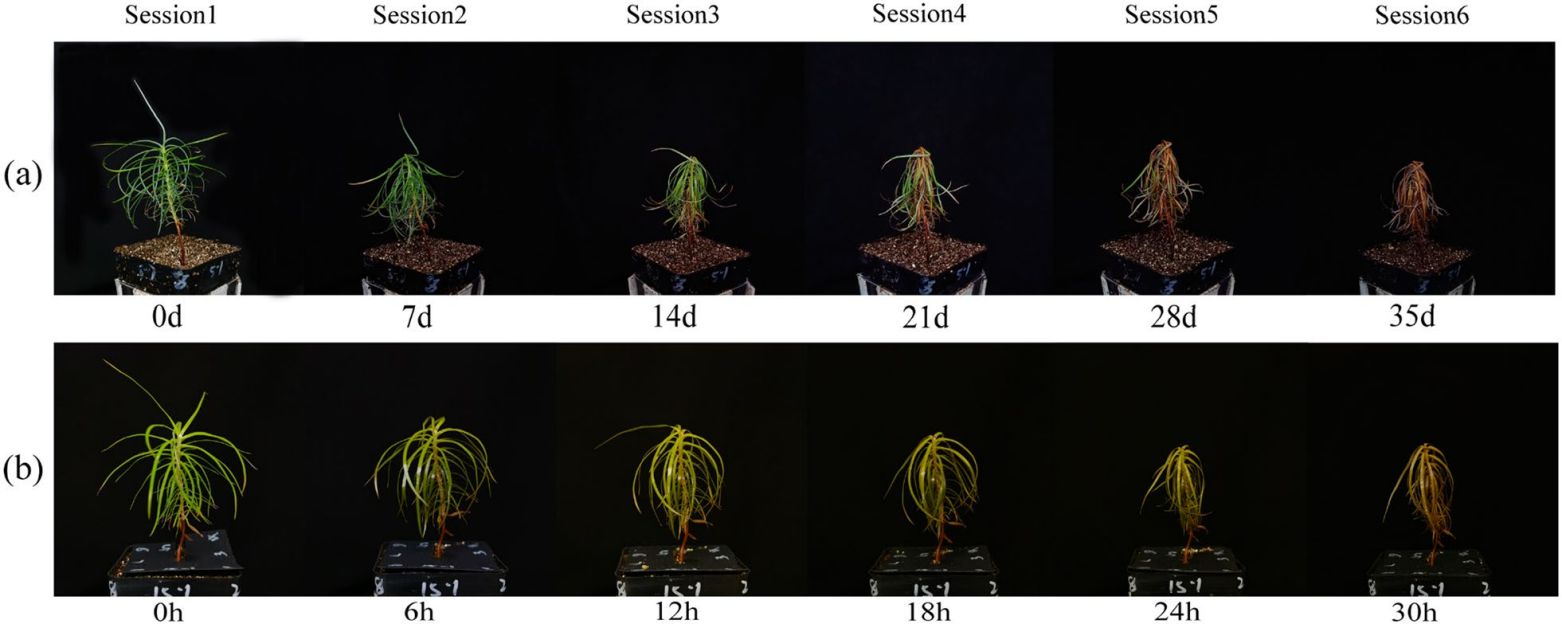
Representative images of the two datasets. **a** Drought stress **b** heat stress

The images were captured by Canon camera (PowerShot SX720 HS, Canon Inc., Tokyo, Japan) in a small photo studio. Images of seedlings under heat stress were photographed at regular intervals of 6 hours, and images of seedlings under drought stress were taken every seven days. For each seedling, images were taken from eight angles at every 45^◦^. In order to ensure the robustness of the classification, we take pictures at a fixed position, so that all images of a dataset were taken from the same angle. The parameters for taking photographs including lighting condition, the camera distance, image size in pixels, and other information were listed in Table 1. Finally, 2424 images (404 images of each session) were captured for the seedlings under drought stress, and 1776 images (296 images of each session) were captured for the seedlings under heat stress. Based on the growth status of seedlings, each image was manually scored with a value from 0 to 1.0 as a label, which was used in loss function. Accordingly, the growth status of seedlings from drought and heat stress was classified into 6 sessions, respectively (Fig. 1). A stratified 5-fold cross-validation approach was utilized to evaluate models. For that, 80% of images were prepared for training, and 20% were taken for the testing. And, 20% of training data was used as a validation set to prevent overfitting problems.

**Table 1:** Parameters used for image acquisition

Treatment	Image size in pixels	Image Type	Total Images	Light used	Distance of camera
Heat	3072 × 3072	RGB(JPEG)	2424	Fluorescent tubes	0.2 m
Drought	3072 × 3072	RGB(JPEG)	1776	Fluorescent tubes	0.2 m

### Deep learning-based feature extraction

Although several pre-trained CNN architectures have been proposed for plant phenotyping [21], selecting the most appropriate CNN architectures for depth feature (DF) extraction is a challenging task. In this study, we used VGG16 [34], AlexNet [35], ResNet18 and ResNet50 [36] for training. All these networks were pre-trained on the ImageNet public dataset to classify the images into 1000 classes. These networks differed in the input size, number of layers, and the number of the learnable parameters. In our study, the last layers of these networks were replaced by a classification layer with 6 neurons to classify images into six sessions. Before training, the RGB image size is adjusted to (448,448,3) to fit different networks. We used transfer learning to fine-tune pre-trained CNNs models on the ImageNet [37] dataset, and then used these models to classify Chinese fir seedlings under heat stress and drought stress, and subsequently used these models as feature extractors for the CNN-LSTM model. Stochastic gradient descent algorithm was applied to optimize the model performance.

### CNN-LSTM architecture

**Fig. 2:**
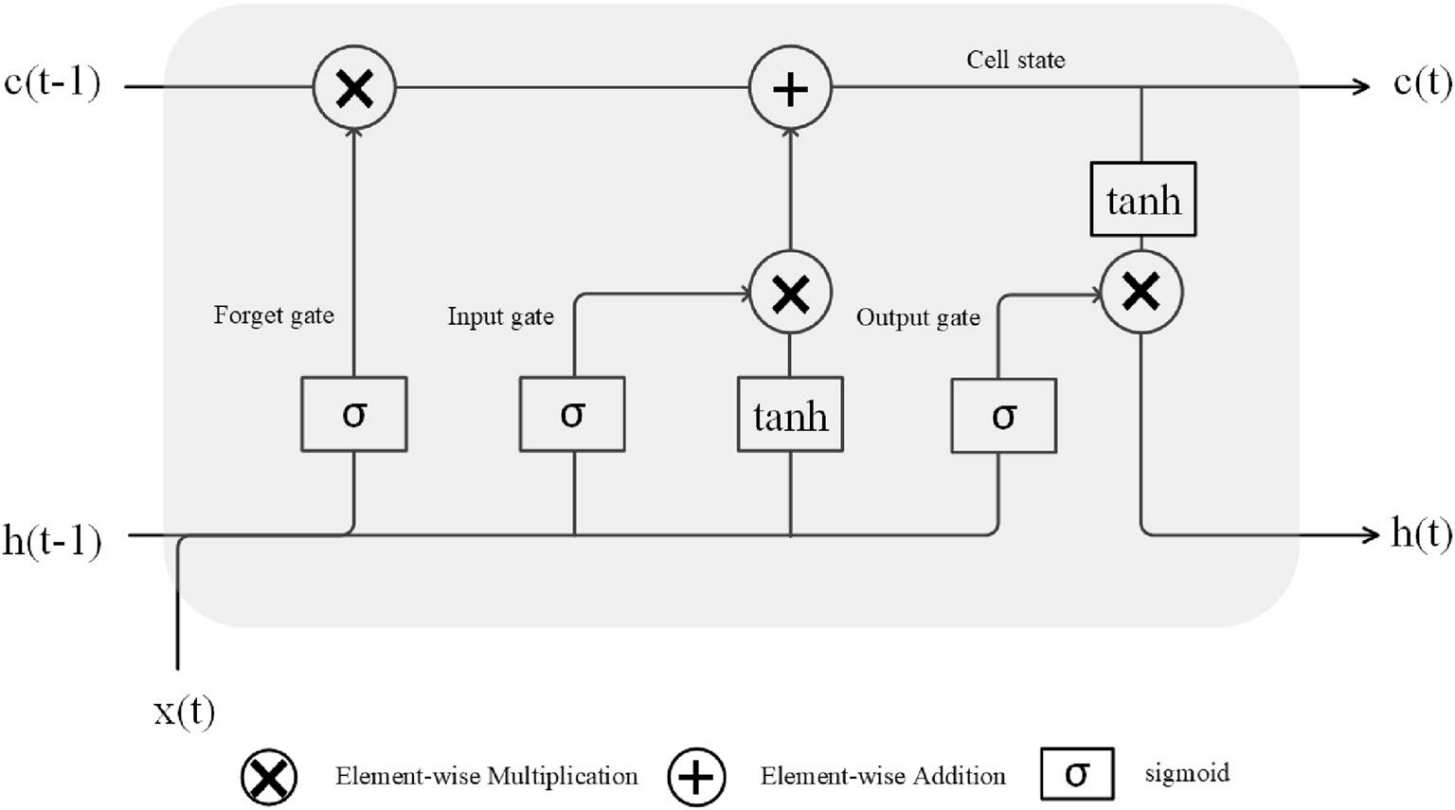
The main components of the LSTM unit

The growth and development of plants are a dynamic process not only related to spatial, but also associated with temporal information, which are not considered in conventional CNN model. As a specialized form of Recurrent Neural Network (RNN) architecture, the LSTM network can learn long-term dependencies and preserve useful temporal information for an extended period [38]. Compared with simple RNN, the LSTM is more suitable for sequential data such as time-series. To date, the LSTM has been widely used in jump shot performance in youth basketball, language modeling, speech recognition and stock price prediction [39–42]. Also, the LSTM was exhibited excellent capabilities in plant growth and development prediction, prediction of diseased rice plant and nutrient status diagnosis of infield oilseed rape [22, 43, 44]. In our study, as presented in Fig. 2, the LSTM was mainly composed of forget gate, input gate, output gate which were used to control the cell state. All these gates connect the input of the current time step(xt) to the hidden state of the previous time step(ht − 1). The forget gate is responsible for deciding which cell states from the previous time step should be preserved. The input gate controls how much of the new input data should be recorded into the cell state. The output gate completes the selective memory, update of the information and outputs the piece of the information using the sigmoid and the tanh.

**Fig. 3:**
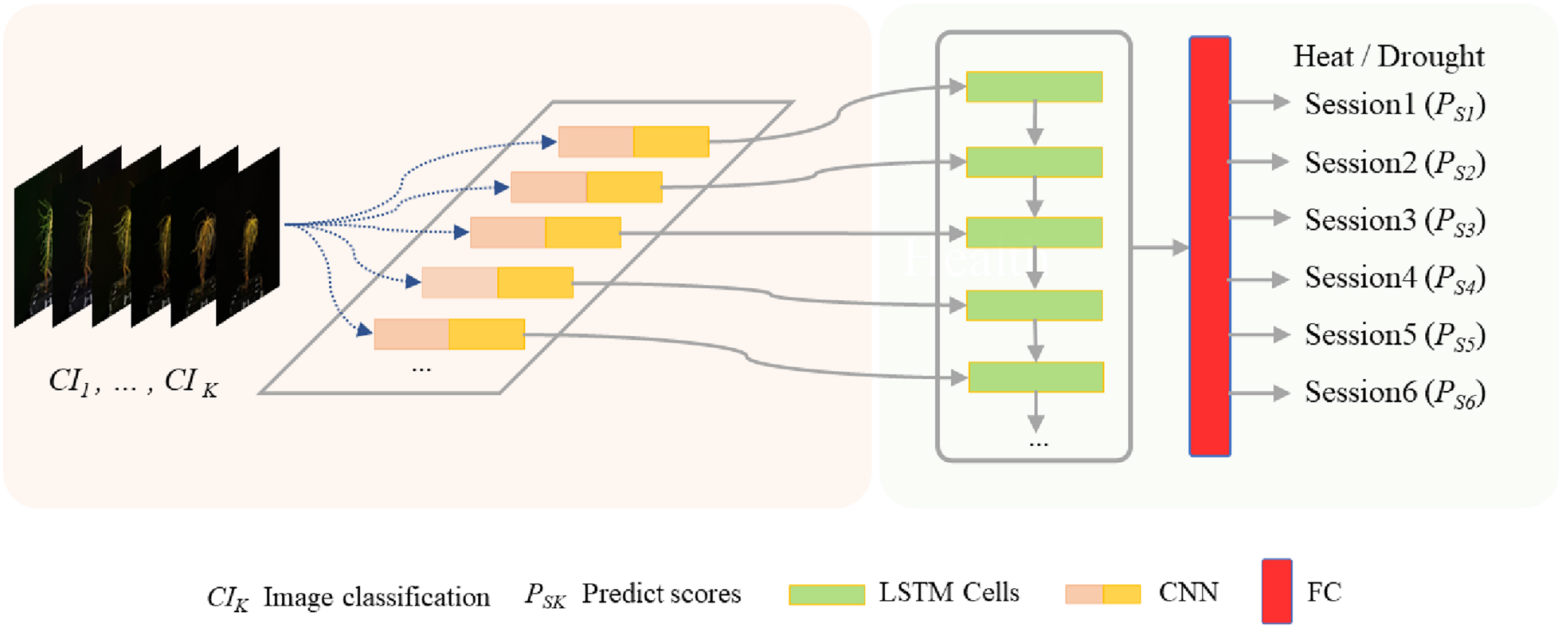
The proposed CNN-LSTM framework for time-series image dataset in our study

CNN-LSTM hybrid model has been successfully used in tasks requiring sequence learning of visual features [45], like video classification and activity recognition in videos [18, 46]. Our task was similar to activity classification in videos that predict which activity is being performed by analyzing visual changes over time. Thus, we proposed a modified CNN-LSTM model to classify growth status of Chinese fir seedlings under heat and drought stress. Our CNN-LSTM architecture for heat and drought stress is shown in Fig. 3. The workflow was briefly described here. At first, the time series dataset and the manual scored value were fed into the CNN model for feature extraction. Then, deep features were extracted from the last fully-connected (FC) layers of the CNN models and fed to the LSTM model. The number of sequentially connected cells is equal to the number of session data used for prediction. The LSTM network output is fed into a fully connected layer of size 512-D, which is connected to the fully connected Layer of size 6, equal to k heat and drought stress. The cross-entropy loss and L2 loss were employed as a loss function, and hyperparameters of the LSTM are presented in Table 2.

**Table 2:** Description of the LSTM architecture hyperparameters

Parameters	Specification
Input gate	Sigmoid
Forget gate	Sigmoid
Output gate	Sigmoid and tanh
Hidden layer	Tanh
Number of layers	1

### Improve CNN-LSTM with attention mechanism

#### Attention-based modules

The attention mechanism in deep learning is similar to visual attention of humans, which selectively focuses on the information that is beneficial to the final result. In our study, attention mechanism was introduced into CNN-LSTM to improve the classification accuracy.

**Fig. 4:**
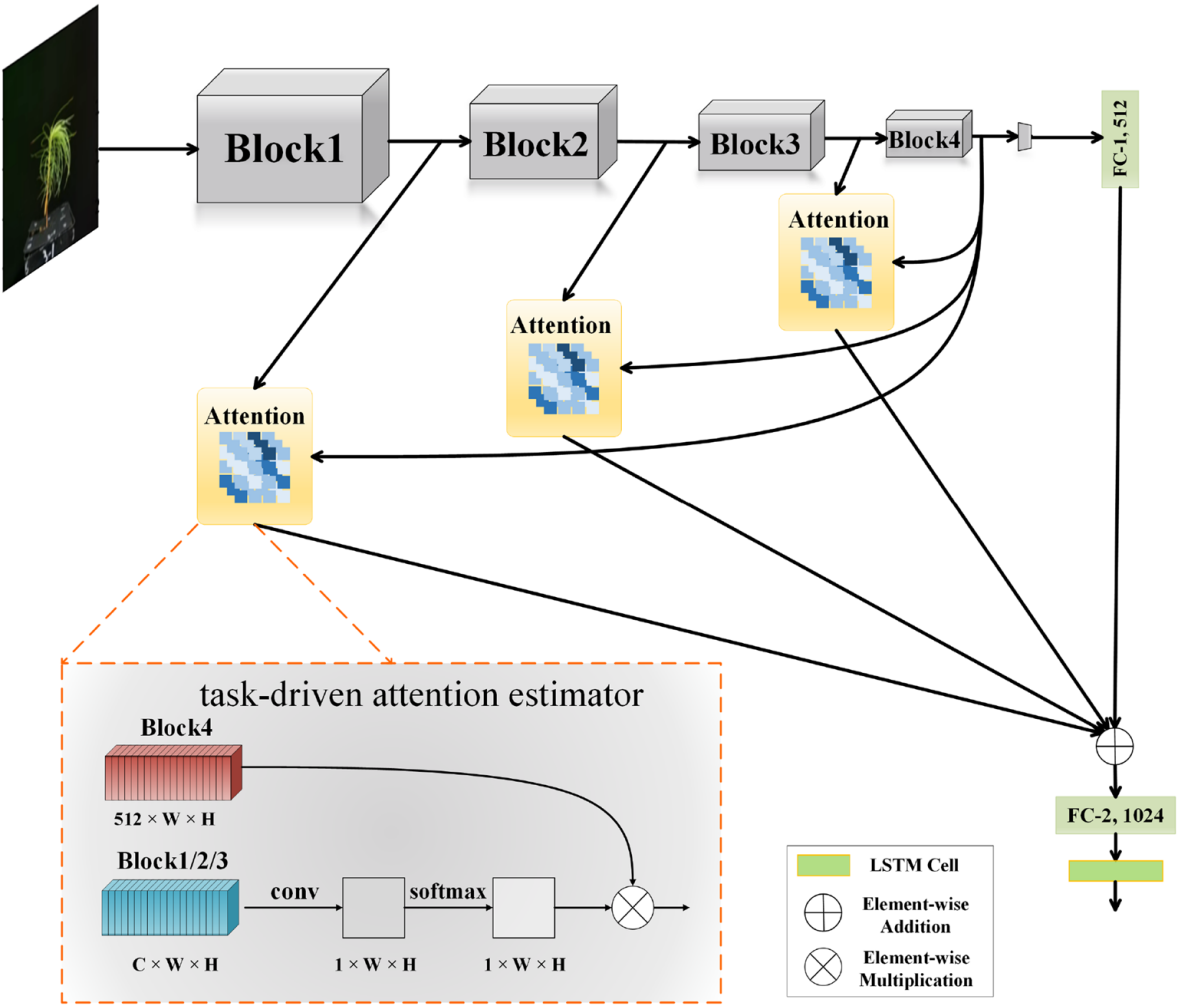
Attention introduced at 3 distinct layers of ResNet50 and the structure of designed task-driven attention estimator

The proposed approach is illustrated in Fig. 4. Block 1/2/3 is the local feature, which is the intermediate feature output at different scales in the ResNet50 network. Block4 is treated as a global feature, which has the entire input image as support and outputs by the network’s series of convolutional and nonlinear layers. Local and global features were fed into the attention mechanism, and the estimator can generate new feature maps instead of local features of the image. Concatenating the output of different local feature maps and Resnet50 last layer as the new output, and the final output is fed into the fully connected layer classifier (FC-2, 1024).

The modified backbone of network replaces the original backbone of network and sends the output result of the fully connected layer into the LSTM cell. A task-driven attention estimator was designed (Fig. 4). Take intermediate features and global features as input, the dimension local information is compressed to 1 by a 1 × 1 convolution kernel and then normalized by softmax operation. The normalized features are then multiplied by the Block4, by element-wise multiplication. By defining a compatibility measure between local and global features, we redesign the standard architecture to classify input images using a weighted combination of local and global features, so the network is forced to learn attention patterns relevant to solving the task at hand.

### Loss function

he loss function denotes the differences between the prediction and the ground truth, which is essential for network training. In this study, cross-entropy loss and L2 loss function were used to train the network. The loss of the network is computed using Eqs. (1)–(3).

1 $$L= {L}_{cls}+{L}_{pro}$$

2 $$L_{{cls}} = - \sum\limits_{{i = 0}}^{n} {c_{i} log\left( {\hat{c}_{i} } \right)}$$

3 $$L_{{pro}} = \frac{1}{n}\sum\limits_{{i = 0}}^{n} {(y_{i} - \hat{y}_{i} )^{2} }$$

where L_cls_ is the loss of classification, L_pro_ is the loss of manual scored value regression. ${c}_{i}$ and ${\widehat{c}}_{i}$ denote the predicted and truth classification. ${y}_{i}$ and ${\widehat{y}}_{i}$ denote the predicted scores and manual scored value.

This study is multi-task learning with regression and classification objectives. Multi-task learning aims to improve learning efficiency. However, the performance of multi-task learning strongly depends on the relative weight between losses of each task. Manually adjusting these weights is a difficult and expensive process [47]. In this study, a principled multi-task deep learning method is adopted to measure multiple loss functions by considering the homoscedasticity uncertainty of each task [48] The homoscedasticity uncertainty is independent of the input and depends on the inherent uncertainty of the task. By transforming the homoscedasticity uncertainty into the weight of the loss, the model can have the ability to dynamically adjust the loss [49]. This allows tasks to simultaneously learn various quantities with different units or scales in both classification and regression settings. Multi-task loss function is defined as follows:

4 $$LOSS\left(W,{\sigma }_{cla}^{2},{\sigma }_{pro}^{2}\right)= \,\frac{1}{{2\sigma }_{cla}^{2}}{L}_{cla}\left(W\right)+ \frac{1}{{2\sigma }_{pro}^{2}}{L}_{pro}\left(W\right)+\text{log}\left({\sigma }_{cla}^{2}\right)+\text{log}\left({\sigma }_{pro}^{2}\right)$$

Parameters ${\sigma }_{cla}^{2}$, ${\sigma }_{pro}^{2}$ correspond to the loss and the data-based adaptive weights of${L}_{cla}$ and ${L}_{pro}$.

### Classification and evaluation criteria

This study intends to use a confusion matrix to classify and evaluate the plant growth status under stresses. The performance of the model was evaluated at the pixel level and target level (plant part). In both cases, the assessment is based on accuracy (Acc), precision (Pr), recall (Re) and F1 scores. The four parameters can be calculated by Eqs. (5)–(8). TP, TN, FP and FN represent true positive, true negative, false positive and false negative respectively; The total number of all states is N.

5 $$Accuracy=\frac{TP + TN}{N} \times 100\%$$

6 $$precision=\frac{TP}{TP + FP} \times 100\%$$

7 $$recall=\frac{TP}{TP + FN} \times 100\%$$

8 $$\text{F}1 \text{s}\text{c}\text{o}\text{r}\text{e}=2 \times \frac{ precision\cdot recall}{precision + recall}$$

The performances of regression models were assessed using the determination coefficient (R^2^) and root mean square error (RMSE), which were calculated by Eqs. (9)–(10).

9 $${R}^{2 }=1- \frac{{\sum _{i}^{n}(yi - \widehat{y}i)}^{2}}{{\sum _{i}^{n}(yi - \stackrel{-}{y}i)}^{2}}$$

10 $$RMSE =\sqrt{\frac{{\sum _{i=1}^{n}(yi - \widehat{y}i)}^{2}}{n}}$$

where $yi$ and $\widehat{y}i$ are the manual scored and predicted values, respectively. $\stackrel{-}{y}i$ is the mean of the measured values, and $n$ is the total number of samples in the testing dataset.

### Experimental setting

The training and testing of the model were performed on an Ubuntu Linux workstation equipped with one Intel Xeon Processor CPU (96 GB RAM) and two Nvidia GeForce RTX 3060Ti graphics cards for acceleration, each with 12 GB of video memory. The model is implemented in the Pytorch 1.12.1 and CUDA 11.3 deep learning open-source framework using Python 3.7. Neural network weights are optimized using Adam optimizer. The initial learning rate, momentum factor and batch size were set to 0.001, 0.9 and 30, respectively, and 300 epochs were trained.

## Results

### Comparison of AlxNet, VGG16, resnet18 and resnet50

At the beginning, we trained and evaluated four CNN models including AlxNet, VGG16, Resnet18 and Resnet50, which were frequently used as feature extractors in plant phenotyping. The results showed that Resnet50 network had the best classification effect on plant images from drought and heat stress (Table 3). When training with Resnet50, for images from heat stress, the Acc, Pr, Re and F1 scores were 77.05%, 76.74%, 76.94% and 76.84%, respectively (Table 3), while for images of drought stress, the Acc, Pr, Re and F1 scores were 75.20%, 75.33%, 75.19% and 75.26%, respectively, (Table 3). It indicated the outperformance of Resnet50 in these CNN models. Resnet50 has more parameters than Alxnet, VGG16 and Resnet18, and the larger the model, the higher the fitting degree, and the better the classification performance for heat and drought stress. On the other hand, better performance of Resnet50 was shown on heat stress images than drought stress images, which was possibly caused by more obvious change in the needle color of Chinese fir seedlings after heat stress. In other words, visual changes brought about by heat stress are more pronounced than drought stress, so it is easier to classify Chinese fir seedlings images after heat stress.

**Table 3:** Performance of the four CNN models in classification of Chinese fir seedlings under drought and heat stress

Stress	CNN Model	Acc (%)	Pr (%)	Re(%)	F1-score (%)
Heat	Alxnet	69.94	69.80	69.90	69.84
	VGG16	69.47	68.87	69.44	69.15
	Resnet18	71.91	71.88	71.77	71.82
	Resnet50	77.05	76.74	76.94	76.84
Drought	Alxnet	70.29	70.44	70.26	70.34
	VGG16	69.06	70.21	69.03	69.61
	Resnet18	71.72	72.11	71.70	71.90
	Resnet50	75.20	75.33	75.19	75.26

### Construction of CNN-LSTM hybrid models

**Fig. 5:**
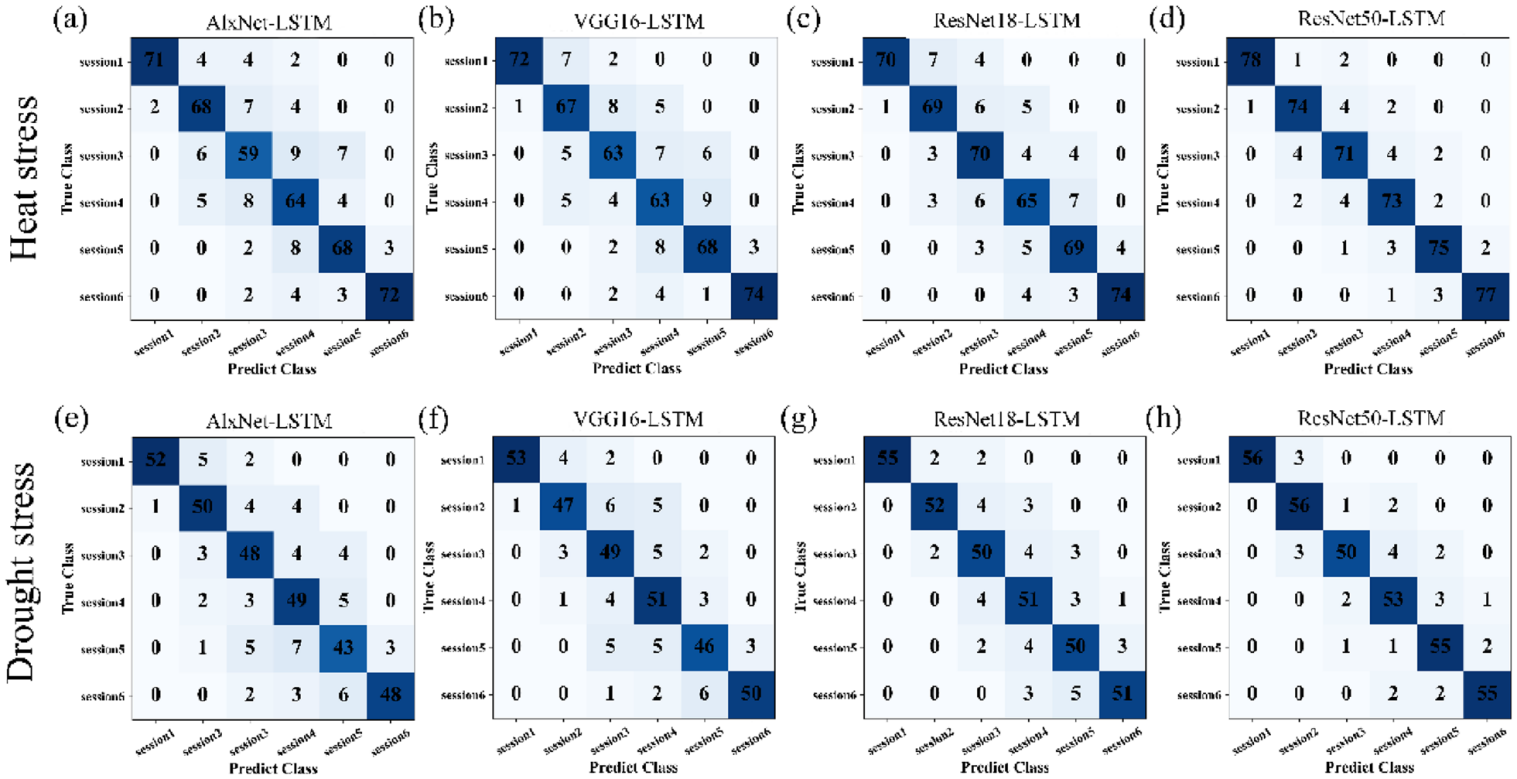
Confusion matrix of classification effects of the four CNN-LSTM models. Heat stress:**a** AlxNet-LSTM, **b**VGG16-LSTM, **c** ResNet18-LSTM and (**d**) ResNet50-LSTM. Drought stress:**e** AlxNet-LSTM, **f** VGG16-LSTM, **g** ResNet18-LSTM and **h**ResNet50-LSTM.

To take the temporal information into consideration, four hybrid models based on above CNN models and LSTM were constructed, and their performances of classification on growth status of Chinese fir seedlings under heat and drought stress were then compared, respectively. As a result, the performances of all four CNN models were improved after conjunction with LSTM (Table 4). Still, Resnet50-LSTM had the best performance. The Acc, Pr, Re and F1-score for images of drought stress reached up to 91.80%, 91.83%, 91.65% and 91.74%, respectively. And, the Acc, Pr, Re and F1-score for images of heat stress reached up to 92.18%, 92.14%, 92.06% and 92.10%, respectively (Table 4). Meanwhile, the confusion matrices also showed that Resnet50-LSTM hybrid model possessed the most powerful ability in classification of growth status for seedlings under drought and heat stress (Fig. 5).

**Table 4:** Performance of the CNN-LSTM models on classification of Chinese fir seedlings under drought and heat stress

Stress	CNN-LSTM Model	Acc (%)	Pr (%)	Re (%)	F1-score (%)
Heat	AlxNet-LSTM	82.72	83.33	82.61	82.95
	VGG16-LSTM	83.74	84.16	83.64	83.90
	Resnet18-LSTM	85.80	86.17	85.69	85.93
	Resnet50-LSTM	92.18	92.14	92.06	92.10
Drought	AlxNet-LSTM	81.92	82.60	81.78	82.19
	VGG16-LSTM	83.61	84.31	83.47	83.89
	Resnet18-LSTM	87.28	87.62	87.14	87.38
	Resnet50-LSTM	91.80	91.83	91.65	91.74

### Resnet50-LSTM versus resnet50-LSTM-att

**Fig. 6:**
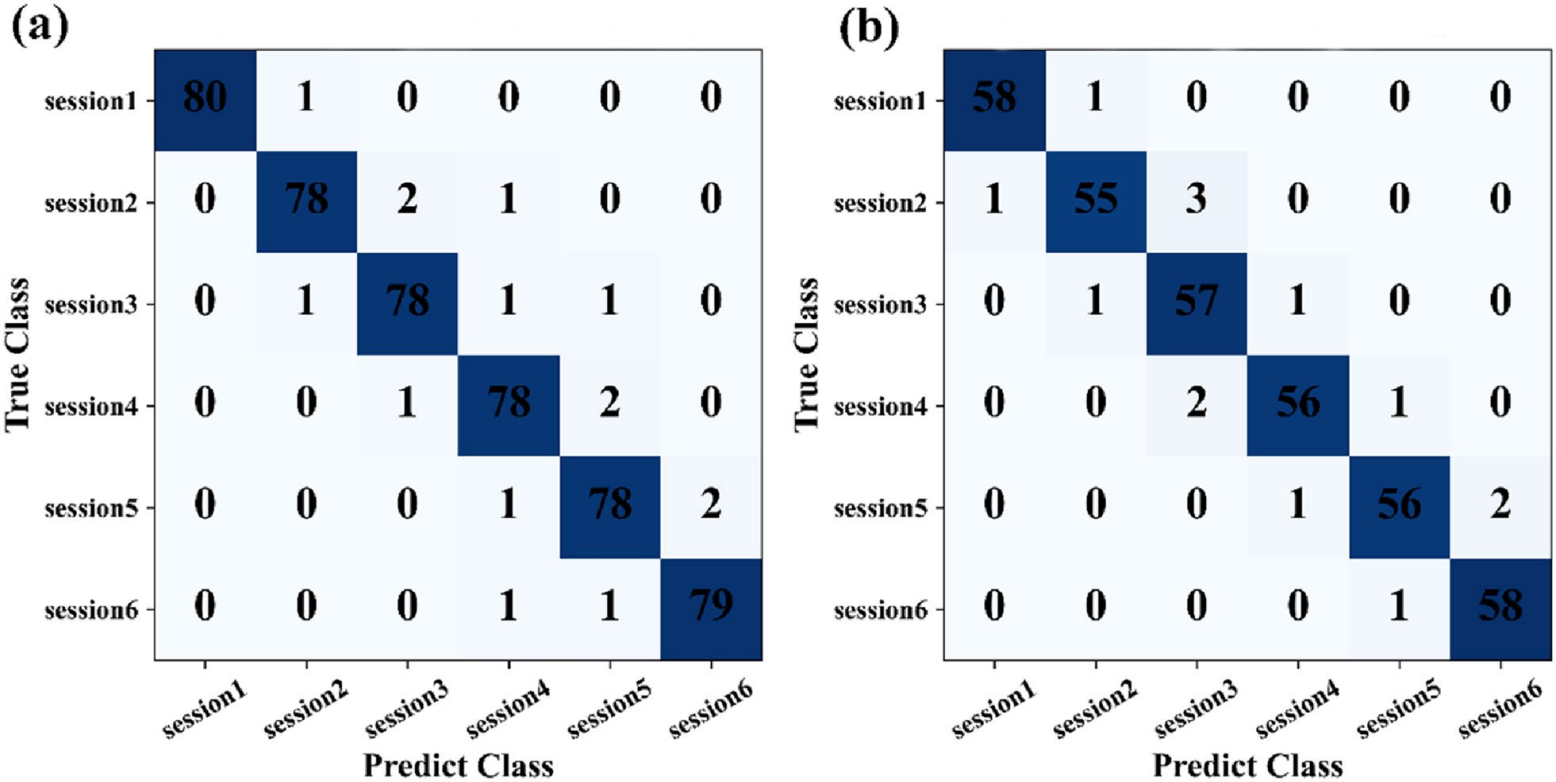
Confusion matrix of the classification effects of Resnet50-LSTM-att on image datasets of **a** heat stress and **b** drought stress

To further improve the CNN-LSTM architecture, attention mechanism (AM) was introduced into Resnet50-LSTM. As shown in Table 5, the introduction of attention mechanism leads to a significant improvement over Resnet50-LSTM in the classification task. Compared with the Resnet50-LSTM model, the performance of Resnet50-LSTM-att in the classification of heat and drought datasets was significantly improved. The Acc, Pr, Re and F1-score of heat stress were 96.91%, 96.81%, 96.79%, and 96.80%, respectively. And, the Acc, Pr, Re and F1-score of drought stress reached 96.05%, 95.92%, 95.88%, and 95.90%, respectively (Table 5). And, the confusion matrix also showed better classification results by Resnet50-LSTM-att (Fig. 6). Obviously, the classification accuracy of the network model is significantly improved after the fusion attention module.

**Table 5:** Performance of Resnet-LSTM model before and after introducing attention mechanism

Stress	Model	Acc (%)	Pr(%)	Re(%)	F1-score (%)
Heat	Resnet50-LSTM	92.18	92.14	92.06	92.10
	Resnet50-LSTM-att	96.91	96.81	96.79	96.80
Drought	Resnet50-LSTM	91.52	91.52	91.37	91.44
	Resnet50-LSTM-att	96.05	95.92	95.88	95.90

### Verification of CNN_attention feature extractor using Grad-CAM

**Fig. 7:**
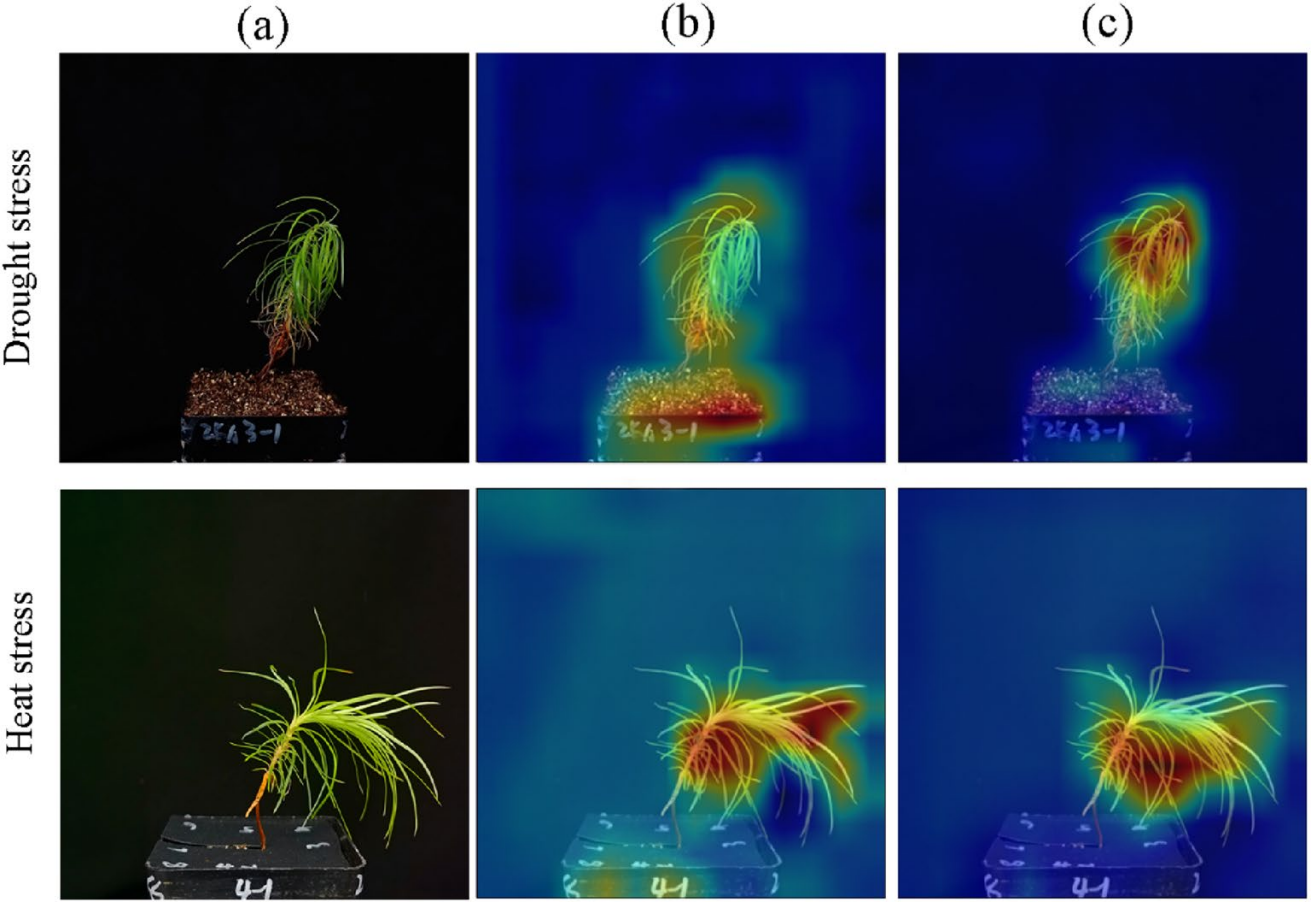
Visualization results of class activation maps before and after adding the attention mechanism. The highlighted part of the class activation map represents the attention of the network on to the image, and the red intensity is proportional to the strength of the neural activation with respect to the predicted class. **a** The original image **b** The region of attention before adding the attention mechanism **c** The region of attention after adding the attention mechanism

Attention mechanism gives more weight to important areas, and pays attention to more differentiated information regions in images, which improves the feature extraction ability for images, thus improving the classification accuracy in our study. Through Grad-CAM, the class activation graph of network layer was visualized before and after attention mechanism introduced into the Resnet50-LSTM model. As shown in Fig. 7, compared with Resnet50-LSTM, Resnet50-LSTM-att network pays more accurate attention to the areas where seedlings located, which means the Resnet50-LSTM-att network gives more weight to the important areas and less weight to the unimportant areas. More specifically, before introducing of AM, the area of the Resnet50-LSTM network attention to in the image included both the seedling and some background regions, which would result in a negative impact on the final classification. After the introduction of the AM, the attention region of the network is more concentrated to the region of Chinese fir seedling inside the image. This explains why AM could improves the accuracy of the final classification in our study.

### Evaluation of growth status by using Resnet50-LSTM-att model

**Fig. 8:**
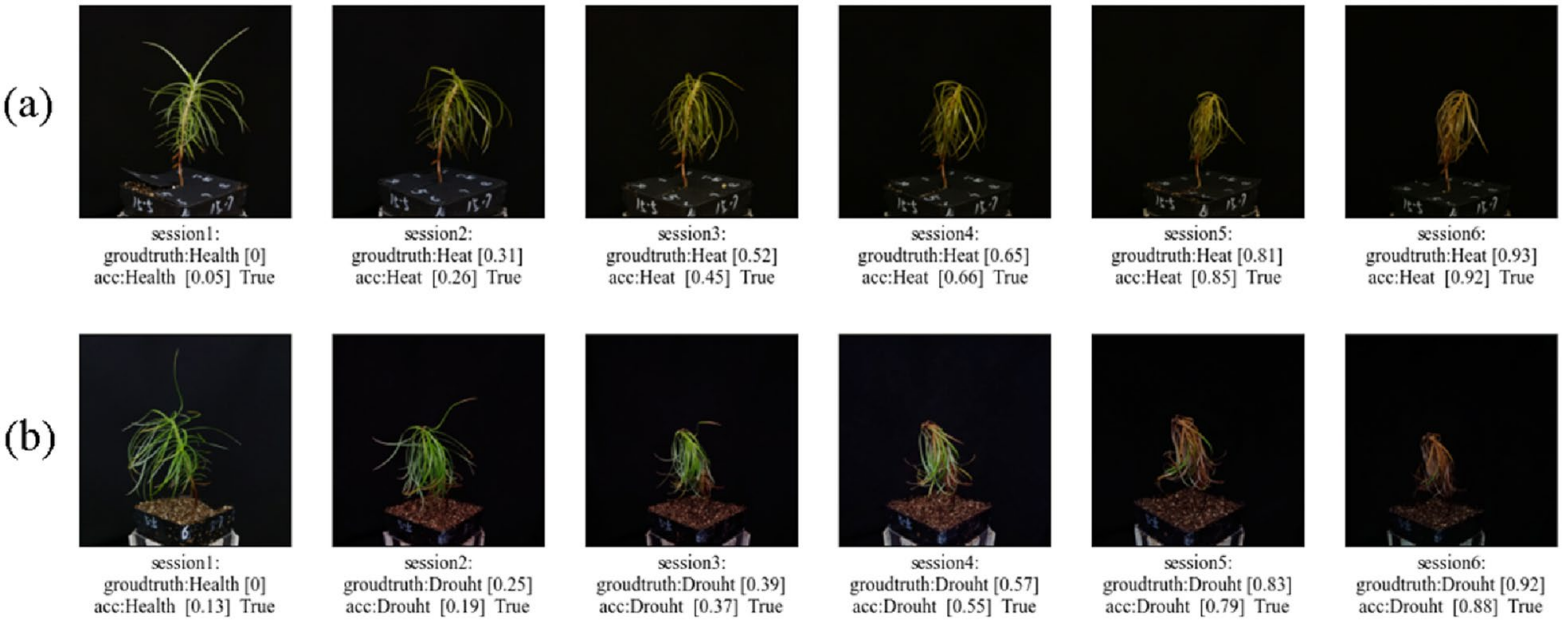
Prediction of the growth status of Chinese fir seedlings from test dataset. **a** Heat stress **b** drought stress

**Fig. 9:**
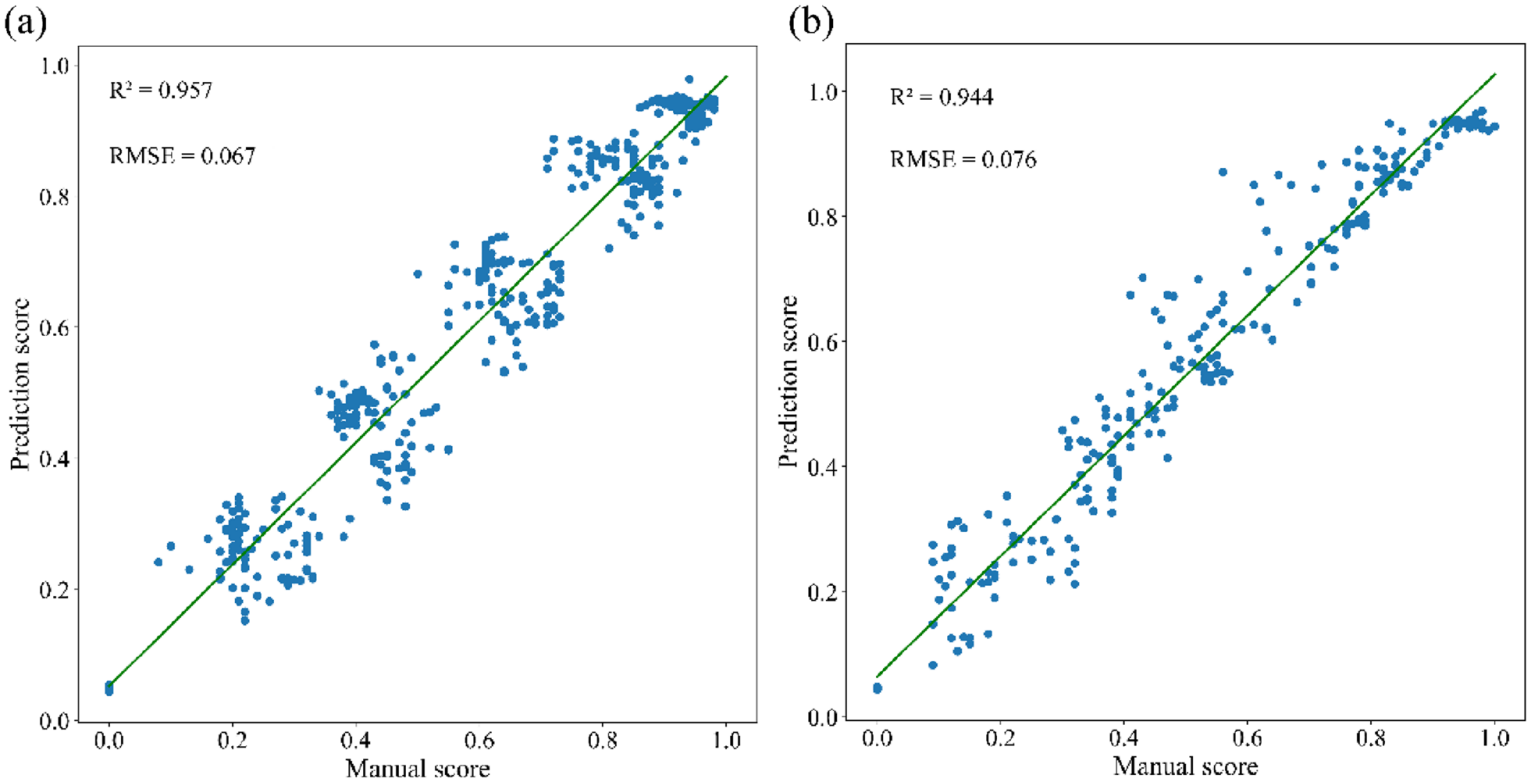
Correlation analysis between manual and prediction scores. **a** Heat stress **b** drought stress

Based on Resnet50-LSTM-att hybrid model, the growth status of seedling from image of test set was evaluated by giving a predict score. As shown in Fig. 8, the growth status of seedlings was successfully evaluated with a prediction score, and classified into six sessions. Correlation analysis showed that the R^2^ and RMSE were 0.957and 0.067 for the dataset of heat stress, respectively, and, R^2^ and RMSE were 0.944and 0.076 for the dataset of drought stress, respectively (Fig. 9). This means that the predicted results were in good agreement with the manual scoring results. According to the predicted score, it is easier to determine the growth status of seedlings. All these results indicated that Resnet50-LSTM-att was the best model for this study. Our framework provides a faster, more convenient and accurate method for classification and evaluation on growth status of Chinese fir seedlings under heat and drought stress. Due to the flexibility of the proposed framework, it also could be utilized in detection and classification of images from different stress conditions in needle-leaved plants.

## Discussion

In past decades, the development of deep learning and image processing provides a great opportunity for their applications in plant phenotyping. Many methods based on different deep learning models have been proposed and applied in yield prediction, disease detection, growth monitoring, nutrient status diagnosis and other tasks in crops and horticultural plants [50–52]. For example, Abdalla et al. [22] proposed an Inceptionv3-LSTM model for automatic nutrient status diagnosis during the whole life cycle of the Oilseed rape. Fan et al. [53] proposed a deep learning framework for segmentation and leaf counting in plant, which achieved good results in *Arabidopsis* and tobacoo plants. Besides, in order to detect and count rice panicle, Wang et al. [54] built a PanicleDetect model based on YOLOv5x, which was proved to be robust and accurate for counting panicles in field images of rice. Similarly, Yu et al. [55] proposed a fast method for soybean disease recognition based on residual attention network (RANet) model. And, Zhou et al. [56] successfully utilized Mask R-CNN to detect bruising on strawberry images captured by color cameras under incandescent light and ultraviolet (UV) light. These studies showed broad applications of deep learning models in recognition, classification and evaluation of phenotypic characteristics in diverse plants.

Chinese fir distributed widely in southern China is one of the main timber trees of plantation in China. In the context of global warming, it has been an important task to select and develop new stress-resistant varieties for breeders of Chinese fir. The main object of this work is to provide a fast, automated and noninvasive method for classification and evaluation on growth status of Chinese fir seedlings under drought and heat stress, which could reduce labor and costs, and raise efficiency and accuracy of breeding works. In previous studies, a prediction model, which based on spatiotemporal long short-term memory (ST-LSTM) and memory network memory (MIM), was proposed to predict the image sequences of future growth and development in wheat [43]. Azimi et al. [57] proposed a deep learning pipeline for the temporal analysis of stress-induced visual changes in plants and applied it to the identification of specific water stress situations in plant shoot images of chickpea. In our study, a hybrid Resnet-LSTM model with AM was designed and constructed for the stress phenotyping of Chinese fir seedlings. Our proposed model could classify the growth status of Chinese fir seedlings based on their images from drought and heat stress, and the model could also accurately evaluate the growth status of the seedling with a prediction value (Fig. 9). Similarly, if sufficient data could be provided, we believe that the proposed model would be feasible for larger seedlings of Chinese fir, other conifers with needle-like leaves, and those stress conditions that can induce similar phenotypic changes. Of course, more data should be collected from seedlings of different sizes and stress conditions to verify the feasibility. In summary, this model would potentially become a powerful tool for breeders to select and develop stress resistant varieties. Meanwhile, by utilizing this model in future, irrigation management in the cultivation of Chinese fir seedlings would probably be more efficient so that more water resources and manpower could be saved.

Advances in phenomics and genomics have brought unprecedented amounts of new data, which requires more intelligent and more efficient tools to deal with. As an important aspect of artificial intelligent (AI), deep learning has merged as a versatile tool in phenotypic analysis and breeding practice. However, in contrast to crops or several important fruit plants, much less applications of deep learning principles have been reported in timber trees. In this study, for the first time, we proposed a CNN-LSTM-att model as a tool for stress phenotyping of Chinese fir seedlings. To ensure the accuracy, robustness and predictive power, two datasets consisting of 2424 and 1776 images were generated to train the model. Interestingly, we found that a sample size of at least 1000 images is required to effectively train the model (data not shown). This means that relatively large amounts of data are still necessary to build a useful deep learning model. On the other hand, it is actually difficult to characterize the phenotypic changes of seedlings under stress conditions with only one or a few indicators. More morphological and physiological indicators should be collected to train such a deep learning model, so that the classification and evaluation by the model would have more biological meaning. Our study is an interesting and meaningful attempt for application of deep learning method in stress phenotyping of Chinese fir. It provides a good reference for similar timber tree, and would help to promote their breeding programs.

## Conclusion

In this study, a hybrid deep learning model Resnet50-LSTM-att was proposed to classify and evaluate the growth status of Chinese fir seedlings under drought and heat stress. Our study showed the importance of introducing time series information to detect the growth status of Chinese fir seedlings. By comparing four base CNN models, Rensnet50 was selected as the backbone network. Conjunction of Resnet50 with LSTM dramatically improves classification accuracy for both image datasets of the Chinese fir seedlings under drought and heat stress. Furthermore, introduction of the attention mechanism, which would drive the Resnet50-LSTM model pay more attention to the region where seedling located inside the image, could greatly improve the performance of the model. By utilizing the Resnet50-LSTM-att model, the accuracy rate, precision rate, recall rate and F1-score of classification on the dataset of heat stress were 96.91%, 96.81%, 96.79%, and 96.80%, respectively. And, the accuracy rate, precision rate, recall rate and F1-score of classification on the dataset of drought stress were 96.05%, 95.92%, 95.88%, and 95.90%, respectively. Accordingly, R2 value and RSME value for evaluation on growth status under heat stress were 0.957 and 0.067, respectively. And, R2 value and RSME value for evaluation on growth status under drought were 0.944 and 0.076, respectively. In conclusion, a Resnet50-LSTM hybrid model with attention mechanism was designed and constructed in our study. This hybrid model is robust and accurate in classification and evaluation of growth status of Chinese fir seedlings under drought and heat stress.

## Data Availability

The datasets used analyzed during the current study are available from the corresponding author on reasonable request.

## References

[1] Allan RP, Masson-Delmotte V (2021). IPCC, 2021: Summary for policymakers in climate change 2021: the physical science basis 2021. Contribution of working group i to the sixth assessment report of the intergovernmental panel on climate change.

[2] Allen CD (2010). A global overview of drought and heat-induced tree mortality reveals emerging climate change risks for forests. For Ecol Manag.

[3] Jing MD et al. Warming-induced drought leads to tree growth decline in subtropics: evidence from tree rings in central China. Front Plant Sci, 2022. 13. 10.3389/fpls.2022.964400 PMC9539437 36212337

[4] Choat B (2012). Global convergence in the vulnerability of forests to drought. Nature.

[5] Wang B (2012). Biomass carbon pools of *Cunninghamia*
*lanceolata* (Lamb.) Hook. Forests in subtropical China: characteristics and potential. Scand J For Res.

[6] Zhou T (2016). Effects of elevated mean and extremely high temperatures on the physio-ecological characteristics of geographically distinctive populations of *Cunninghamia lanceolata*. Sci Rep.

[7] Liu L (2019). Impact of initial planting density on the optimal economic rotation of chinese fir (Cunninghamia lanceolata (Lamb.) Hook) in an experimental forest plantation. Forests.

[8] Bian F (2021). Drought stress introduces growth, physiological traits and ecological stoichiometry changes in two contrasting Cunninghamia lanceolata cultivars planted in continuous-plantation soils. BMC Plant Biol.

[9] Li M et al. Mitigation effects of exogenous acetic acid on drought stress in Cunninghamia lanceolata. Plant and Soil. 2022; 1–16.

[10] Dong TF (2016). Growth, biomass allocation and photosynthetic responses are related to intensity of root severance and soil moisture conditions in the plantation tree *Cunninghamia lanceolata*. Tree Physiol.

[11] Kershaw JA, Larsen DR (1992). A rapid technique for recording and measuring the leaf area of conifer needle samples. Tree Physiol.

[12] Patrício DI, Rieder R (2018). Computer vision and artificial intelligence in precision agriculture for grain crops: a systematic review. Comput Electron Agric.

[13] Singh A (2021). Challenges and opportunities in machine-augmented plant stress phenotyping. Trends Plant Sci.

[14] Jiang Y, Li C. Convolutional neural networks for image-based high-throughput Plant Phenotyping: a review. Plant Phenom. 2020;4152816. 10.34133/2020/4152816 PMC7706326 33313554

[15] Lin K (2019). Deep learning-based segmentation and quantification of cucumber powdery mildew using convolutional neural network. Front Plant Sci.

[16] Selvam L, Kavitha P. Classification of ladies finger plant leaf using deep learning. J Ambient Intell Humaniz Comput, 2020; p. 1–9.

[17] Gong B (2020). Real-time detection for wheat head applying deep neural network. Sens.

[18] Rong J, Chen Y, Yang J (2021). CNN-LSTM Hybrid model for kinematic feature analysis and parabolic radian prediction in basketball videos. Comput Intell Neurosci.

[19] Quan R (2021). Holistic LSTM for pedestrian trajectory prediction. IEEE Trans Image Process.

[20] Guo H, Sung YJS (2020). Movement estimation using soft sensors based on Bi-LSTM and two-layer LSTM for human motion capture. Sens.

[21] Taghavi Namin S (2018). Deep phenotyping: deep learning for temporal phenotype/genotype classification. J Amb Intel Hum Comp.

[22] Abdalla A (2021). Nutrient status diagnosis of infield oilseed rape via deep learning-enabled dynamic model. IEEE Trans Industr Inf.

[23] Turkoglu M, Hanbay D, Sengur A (2019). Multi-model LSTM-based convolutional neural networks for detection of apple diseases and pests. J Ambient Intell Humaniz Comput.

[24] Chang L (2021). Using a hybrid neural network Model DCNN–LSTM for Image-based nitrogen nutrition diagnosis in Muskmelon. Horticulturae.

[25] Yang L (2022). Real-time classification of invasive plant seeds based on improved YOLOv5 with attention mechanism. Diversity.

[26] Zhang M, Su H, Wen J (2021). Classification of flower image based on attention mechanism and multi-loss attention network. Comput Commun.

[27] Jetley S, et al. Learn to pay attention. arXiv:1804.02391. 2018.

[28] Vaswani A (2017). Attention is all you need. ArXiv.

[29] Zhang B (2020). Neural machine translation with GRU-Gated attention model. IEEE Trans Neural Netw Learn Syst.

[30] Zeng W, Li MJC, Agriculture Ei (2020). Crop leaf disease recognition based on self-attention convolutional neural network. Comput Electron Agric.

[31] Zheng CW (2020). Deep learning for strawberry canopy delineation and biomass prediction from high-resolution images. Plant Phenomics.

[32] Shoaib M (2022). Deep learning-based segmentation and classification of leaf images for detection of tomato plant disease. Front Plant Sci.

[33] Minowa Y, Kubota Y (2022). Identification of broad-leaf trees using deep learning based on field photographs of multiple leaves. J For Res.

[34] Karen S, Andrew Z (2014). Very deep convolutional networks for large-scale image recognition arxiv - cs - computer vision and pattern recognition. Computer Science.

[35] Krizhevsky A, Sutskever I (2017). E.J.C.o.t.A. Hinton, *Imagenet classification with deep convolutional neural networks*. Commun ACM.

[36] He K et al. Deep residual learning for image recognition 2016 ieee conference on computer vision and pattern recognition (CVPR), 2015: 770–778.

[37] Guillaumin M, Kuttel D, Ferrari V (2014). Imagenet auto-annotation with segmentation propagation. Int J Comput Vision.

[38] Hochreiter S, Schmidhuber J (1997). Long short-term memory. Neural Comput.

[39] França C (2021). The jump shot performance in youth basketball: a systematic review. Int J Environ Res Public Health.

[40] Greff K (2017). LSTM: a search space odyssey. IEEE Trans Neural Netw Learn Syst.

[41] Banik S (2022). LSTM based decision support system for swing trading in stock market. Knowl Based Syst.

[42] Kim J, El-Khamy M, Lee J. Residual LSTM: design of a deep recurrent architecture for distant speech recognition. arXiv. 10.48550/arXiv.1701.03360.

[43] Wang CY (2022). Predicting plant growth and development using time-series images. Agronomy.

[44] Verma T, Dubey S (2021). Prediction of diseased rice plant using video processing and LSTM-simple recurrent neural network with comparative study. Multimed Tools Appl.

[45] Bao T (2020). A CNN-LSTM hybrid model for wrist kinematics estimation using surface electromyography. IEEE Trans Industr Inf.

[46] Ullah A (2018). Action Recognition in Video sequences using deep bi-directional LSTM with CNN features. Ieee Access.

[47] Kokkinos I. Ubernet: Training a universal convolutional neural network for low-, mid-, and high-level vision using diverse datasets and limited memory. 30th IEEE/CVF Conference on Computer Vision and Pattern Recognition (CVPR), 2017: 6129–6138.

[48] Kendall A, Gal Y, Cipolla R. Multi-task learning using uncertainty to weigh losses for scene geometry and semantics. 2018 IEEE/CVF Conference on Computer Vision and Pattern Recognition (CVPR), 2018: 7482–7491.

[49] Kendall A. and Y.J.A.i.n.i.p.s. gal, what uncertainties do we need in bayesian deep learning for computer vision. NIPS. 2017; 30.

[50] Singh AK (2018). Deep learning for plant stress phenotyping: trends and future perspectives. Trends Plant Sci.

[51] Araus JL (2022). Crop phenotyping in a context of global change: what to measure and how to do it. J Integr Plant Biol.

[52] Yang B, Xu YJHR (2021). Applications of deep-learning approaches in horticultural research: a review. Hortic Res.

[53] Fan X (2022). A segmentation-guided deep learning framework for leaf counting. Front Plant Sci.

[54] Wang X (2022). Field rice panicle detection and counting based on deep learning. Front Plant Sci.

[55] Yu M (2022). A recognition method of soybean leaf diseases based on an improved deep learning model. Front Plant Sci.

[56] Zhou X (2022). Deep learning-based postharvest strawberry bruise detection under UV and incandescent light. Comput Electron Agric.

[57] Azimi S, Wadhawan R, Gandhi TK (2021). Intelligent monitoring of stress Induced by water deficiency in plants using deep learning. Ieee Trans Instrum Meas Ieee T Instrum Meas.

